# The impact of quality-of-life data in relative effectiveness assessments of new anti-cancer drugs in European countries

**DOI:** 10.1007/s11136-017-1574-9

**Published:** 2017-04-11

**Authors:** Sarah Kleijnen, Teresa Leonardo Alves, Kim Meijboom, Iga Lipska, Anthonius De Boer, Hubertus G. Leufkens, Wim G. Goettsch

**Affiliations:** 1 Heath Care Department, Zorginstituut Nederland, Eekholt 4, 1112 XH Diemen, The Netherlands; 2 Division of Pharmacoepidemiology and Clinical Pharmacology, Faculty of Science, Utrecht Institute for Pharmaceutical Sciences, Utrecht, The Netherlands; 3 VU University Amsterdam, Amsterdam, The Netherlands; 4 Center for Innovation in Regulatory Science, London, UK

**Keywords:** Comparative effectiveness, Quality of life, Health technology assessment, Reimbursement, Antineoplastic agents, Patient-centred outcome research

## Abstract

**Purpose:**

The aim of this study is to investigate the role of health-related quality-of-life (QoL) data in relative effectiveness assessments (REAs) of new anti-cancer drugs across European jurisdictions, during health technology assessment procedures.

**Methods:**

Comparative analysis of guidelines and publicly available REAs in six European jurisdictions of anti-cancer drugs approved by EMA between 2011 and 2013.

**Results:**

Fourteen anti-cancer drugs were included, adding up to 79 REAs. Whilst all guidelines state that QoL is a relevant endpoint to determine the relative effectiveness of new cancer drugs, QoL data were included in only 54% of the 79 reports and their impact on the recommendations was limited.

**Conclusions:**

Whilst national guidelines recognize the relevance of QoL to determine the relative effectiveness of new anti-cancer drugs, this is not well-reflected in current assessments. Developing and implementing into REAs specific evidence requirements for QoL data would improve the use of this patient-centred outcome in future reimbursement and pricing decisions.

**Electronic supplementary material:**

The online version of this article (doi:10.1007/s11136-017-1574-9) contains supplementary material, which is available to authorized users.

## Introduction

As the aim of anti-cancer therapies is to allow patients to live better and/or longer, treatment outcomes showing improvements in patient survival (e.g. overall survival) and/or health-related quality of life (QoL) are central to determine the clinical meaningfulness of a new treatment [1].

Health-related QoL can reflect a patient’s day-to-day functioning [2], and is defined as the patient’s subjective perception of his or hers physical, psychological, social, somatic functioning and general well-being [3]. Health-related QoL is particularly relevant in diseases such as cancer that greatly affect all dimensions of daily life [4], as it can convey (additional) information to assess the overall burden of disease, the effectiveness and side effects of the treatment [5]. For example, QoL data can be very informative in advanced disease stages when survival differences are expected to be minimal and treatment-related toxicity is of interest and/or one of the treatments is expected to be more palliative than the others [6]. In addition, QoL data can help understand the impact of novel treatment on patient functioning and to identify treatment-related symptoms that need management [7].

Over the years there has been a growing discussion on how to define and measure health-related QoL in cancer [5]. A patient’s QoL is usually measured through self-completion of validated questionnaires, which can be subdivided into generic- and disease-specific instruments. Most QoL measures are multidimensional, designed to reflect multiple domains of impact. These vary by instrument, but often include physical, psychological and social components of outcome [5]. Examples of commonly used disease-specific questionnaires in cancer research are the “European Organization for the Research and Treatment of Cancer Quality of life Questionnaire” (EORCT-QLQ) and the “Functional Assessment of Cancer Therapy” (FACT). These questionnaires mainly express QoL in terms of tumour-, treatment- and symptom-specific scores by asking patients to answer questions about, for instance, side-effects or discomfort [8, 9]. Commonly used generic QoL instruments in cancer research, on the other hand, are the EuroQol (EQ-5D) and its visual analogue subscale (EQ-VAS). The EQ-5D measures mobility, self-care, usual activities, pain/discomfort and anxiety/depression at three levels of response, while the EQ-VAS represents health status on a scale from 0 (worst imaginable health state) to 100 (best imaginable health state) [10].

Whereas disease-specific QoL data may be more sensitive to detect changes in disease-related symptoms and patient functioning, generic instruments are particularly important to ensure coherence when assessing health benefits across different interventions and multiple indications as they encompass all dimensions relevant to patients, not only those on which an effect is expected [11]. Therefore, both instruments are often seen as complementary.

Although the value of QoL data is evident, there are considerable challenges with collecting and interpreting such data [3, 12]. QoL data collection is time consuming for advanced cancer patients who are hardly able to fulfil the requirements of intensive patient participation. Consequently, data are often incomplete or lacking, making it difficult to identify meaningful effects of treatments on QoL. In addition, the interpretation of health-related QoL evidence is often a challenge as its assessment is, by definition, subjective and problematic to generalize between different patient populations and countries [12]. Another methodological constraint is the fact that oncology trials are frequently open label and information bias becomes a concern [5].

At regulatory level, patient-centred outcomes have been recognized as relevant by the European Medicines Agency (EMA) [13, 14]. To a large extent, such acceptance has been fuelled by clinicians, patients and caregivers [15–17]. Two different studies found that one-third of the EMA reports included patient reported measures among which QoL data, with the latter being more frequently mentioned in reports of antineoplastic agents [18, 19]. A similar trend is observed at the Food and Drug Administration (FDA) where a draft guidance on the use of patient-reported outcomes in industry-sponsored studies was released for public consultation by the FDA in early 2006 and later updated in 2009 [20].

On the pathway for patient access to new drugs, regulatory approval is the first step. Within the European Union, a successful marketing authorization is generally followed by a myriad of health technology assessments (HTAs) at the national level guiding pricing and/or reimbursement recommendations. A relative effectiveness assessment (REA) of a new drug is a particular type of HTA that compares the clinical benefit of a drug with standard treatment. In many European countries, it is a relevant criterion in pricing and/or reimbursement decisions [21]. Previous studies have shown that QoL is considered a relevant endpoint in relative effectiveness assessments (REAs) of new drugs [22]. On the other hand, there have also been reports about a lack of consensus on which QoL data are to be used [11], indicating that challenges exist in this domain. The aim of this study is to investigate whether the perceived importance of QoL data is reflected in REAs for pricing and/or reimbursement recommendations for oncology drugs in Europe. We want to investigate the relevance of QoL data in European REAs by answering the following questions:

- Which requirements are included in methodological guidelines of different EU jurisdictions on the use of QoL data in REAs?
- Are QoL data included in the REAs of new cancer drugs across different EU jurisdictions? If so, how do they impact the recommendations?
- Are there differences in the use of different types of QoL instruments and how do these affect the recommendations?

## Methods

### Research design

We have conducted a retrospective comparative cross-sectional analysis of publicly available assessments produced by HTA bodies on anti-cancer medicines authorized in the EU between 2011 and 2013. The data presented in this article are part of a larger study on the use of endpoints in REAs of anti-cancer drugs [23].

For this article, the data collection focused on the use of QoL data in the assessments and their impact on the recommendation.

### Inclusion criteria

#### HTA jurisdictions

We searched for publicly available reports from HTA bodies involved in drug assessment for pricing and reimbursement decisions in jurisdictions within the EU. Reports were publicly available for nine out of the 29 jurisdictions.^1^ From these nine, three were excluded due to insufficient data: Belgium did not publish all the reports they produced, whereas Portugal and Ireland only published brief summaries thus preventing appropriate data extraction and analysis.

Six jurisdictions and their HTA agencies were included in our study:

- England (EN)—National Institute for Health and Care Excellence (NICE);
- France (FR)—Haute Autorité de Santé (HAS);
- Germany (GE)—Institut für Qualität und Wirtschaftlichkeit im Gesundheitswesen (IQWIG);
- The Netherlands (NL)—Zorginstituut Nederland (ZIN);
- Poland (PO)—Agencji Oceny Technologii Medycznych i Taryfikacji (AOTMiT) and
- Scotland (SC)—Scottish Medicines Consortium (SMC).

#### HTA guidelines

National HTA guidelines for medicines’ assessment were obtained from relevant HTA agencies’ websites. If no guideline was available, grey literature was searched to obtain information on the favoured endpoints in the REAs of anti-cancer medicines. Information on QoL data was retrieved with a special focus on REA sections (and not cost-effectiveness sections).

#### Anti-cancer medicines and reports

A list of all new anti-cancer drugs approved by the EMA between 1 January 2011 and 31 December 2013 (*n* = 26) was compiled. We then selected those medicines for which ≥4 HTA reports had been published by different HTA bodies by April 2015 (*n* = 14).

Reassessments for the same indication (due to changes in price or clinical data availability) were excluded. A total of 72 HTA reports were identified. When an HTA report included separate evaluations and/or recommendations for specific (sub)indications, each (sub)indication was included as an item. The 12 IQWIG reports included a total of 25 (sub)indications with separate recommendations. However, for 7 out of the 25 (sub)indications, data were missing and therefore were excluded from our dataset, resulting in a total of eighteen assessments for Germany. One HAS report included 2 (sub)indications with separate recommendations. The final dataset included 79 HTAs assessments. A detailed flow chart of the selection process is provided in Kleijnen et al. [23].

### Data collection and extraction

A structured data collection form was developed and used to extract data from the assessments. The detailed description of the development including validation is described elsewhere [23]. This article focuses on a subset of the questionnaire, which is related to the inclusion of QoL data and their impact on the recommendation (Questions 22–25).

Since our focus was on the REAs and not cost-effectiveness, data were extracted from the reports’ clinical sections and from the overall recommendations. QoL data were defined as any data measured with validated QoL instruments.

**Fig. 1:**
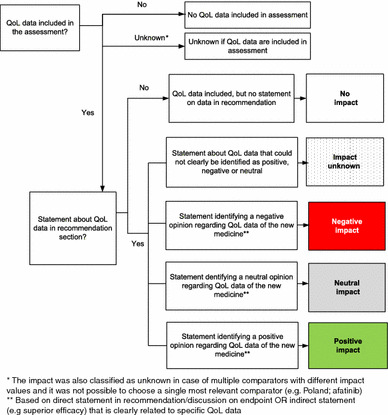
Algorithm used to determine the impact of QoL data on recommendation

In order to capture the impact of QoL data on the recommendation, statements about QoL data in the recommendation/discussion sections of the assessments were categorized as *positive, neutral, negative, unknown* or *no impact* (not identified). The algorithm for QoL data impact categorization is presented in Fig. 1.

Data were collected between April and May 2015 by four researchers, with data abstraction being conducted by a researcher fluent in the jurisdiction’s language.

To improve consistency among researchers’, frequently used statements were identified. In addition, a quality control was conducted by the first author (i.e. checking eventual errors and overall uniformity). Any disagreements were discussed until consensus was reached among all researchers. Furthermore, an expert panel was invited to clarify pending issues. This panel was composed of six experts (one per agency) who are or have been involved in drug assessments. Their review resulted in changes to the categorisation. We initially presumed that an explicit statement about the absence of QoL data impacted negatively on the recommendation. But based on the input from the experts we changed this into ‘no impact'.

### Data analysis

Descriptive statistics were used to summarize the following data and to calculate: the percentage of assessments that included QoL data by jurisdiction, drug and instrument type; and the percentage of statements about included QoL data that were classified as positive, neutral, negative, unknown or no impact across the various jurisdictions and also per type of instrument used. Moreover, data and statements were analysed qualitatively to identify commonalities and disparities across jurisdictions.

## Results

### HTA guidelines

For five out of six jurisdictions, HTA guidelines were identified including information on the use of endpoints in drug assessment. No guideline was identified for France but information was retrieved from a published consensus statement and a review of European countries. Table 1 includes the most relevant information on QoL extracted from the guidelines.

**Table 1:** Overview of information provided on Quality of Life in guidelines of Health Technology Assessment agencies. *Sources* England: National Institute for health and Care Excellence (NICE). Guide to the methods of technology appraisal 2013. Process and methods guide. 4 April 2013. Available from: http://publications.nice.org.uk/pmg9; France: Rima de Sahb-Berkovitch et al. Assessing Cancer Drugs for Reimbursement: Methodology, Relationship between Effect Size and Medical Need. Thérapie 2010 Juillet-Août; 65 (4): 373–377 & Kleijnen S, Goettsch W, d’Andon A, et al. EUnetHTA JA WP5: Relative Effectiveness Assessments (REA) of Pharmaceuticals. Background review. July 2011 (version 5B); Germany: Institute for Quality and Efficiency in Health Care (IQWIG). General Methods. Version 4.1 of 28 November 2013. Available from: https://www.iqwig.de/download/IQWiG_General_Methods_Version_%204-1.pdf; The Netherlands: Zorginstituut Nederland. Dutch Assessment Procedures for the Reimbursement of Outpatient Medicines. Joint publication of the Ministry of Health, Welfare, and Sport and CVZ. March 1, 2010. Available from: http://www.zorginstituutnederland.nl/Dutch_assessment_Procedures_for_the_Reimbursement_of_Outpatient_Medicines.pdf; Poland: Agency for Health Technology Assessment. Guidelines for conducting Health Technology Assessment (HTA). Version 2.1. Warsaw, April 2009. Accessed from: http://www.aotm.gov.pl/www/assets/files/wytyczne_hta/2009/Guidelines_HTA_eng_MS_29062009.pdf; Scotland: Scottish Medicines Consortium. Guidance to Manufacturers for Completion of New Product Assessment Form (NPAF) (October 2014). Accessed from: https://www.scottishmedicines.org.uk/Submission_Process/Submission_guidance_and_forms/Templates-Guidance-for-Submission/Templates-Guidance-fkor-Submission

Jurisdiction	England/Wales	France^a^	Germany	The Netherlands	Poland	Scotland
HTA organization	NICE	HAS	IQWIG	ZIN	AOTMiT	SMC
Separate REA analysis	No, part of cost-effectiveness analysis	Yes	Yes	Yes	No, part of cost-effectiveness analysis	No, part of cost-effectiveness analysis
Patient relevant endpoints	Survival or health-related quality of life	Mortality, morbidity, health-related quality of life	Mortality, morbidity or health-related quality of life	Morbidity, mortality and/or quality of life	Deaths, cases or recoveries, quality of life and adverse effects	Mortality, survival, incidence of disease, morbidity, functional performance, quality of life
Specific guidance on quality of life	For the cost-effectiveness analyses health effects should be expressed in QALYs EQ-5D is the preferred measure of health-related quality of life in adults. QOL data should be reported directly by patients and/or carers Valuation of QoL data should be representative sample of the UK population. The committee finds it helpful to have the perspective of patients or carers about how relevant the clinical outcomes and the standardized generic instruments for measuring health-related quality of life are to the disease or condition.	The Commission often bemoans the lack of quality-of-life data: tools are available in oncology but are difficult to use repeatedly in clinical trials and vary inter-individually, which reduces their relevance for deciding between treatments and therapeutic strategies. As cancers become chronic, other tools such as examining patient preference or utility could be taken into account by the Transparency Commission in oncology.	Use instruments that are suitable for use in clinical trials. RCTs are best suited to demonstrate an effect. If not possible, other efforts are required to minimize and assess bias (e.g. blinded documentation and assessment of outcomes). For particularly serious or even life-threatening diseases, it is usually not sufficient only to demonstrate an improvement in quality of life if at the same time it cannot be excluded with sufficient certainty that serious morbidity or even mortality are adversely affected to an extent no longer acceptable.	Very little research is undertaken that explicitly focuses on quality of life. However, the added value of a medicine may actually be expressed in the form of an improved quality of life…Firm conclusions cannot always be determined based on the Dutch results of research in which quality of life is a secondary parameter.	A cost-utility analysis should be used when: the health-related quality of life is one of the significant outcomes or if the compared technologies give very different clinical effects and it is necessary to find a common denominator. It is admissible to perform the quality-of-life measurement in the patient population or the preference measurement in the general population. The preference measurement for utility assessment is possible by using direct or indirect preference measuring methods. It is recommended to use indirect methods for preferences measurement—validated questionnaires in Polish. While measuring preferences with the EuroQol (EQ-5D) questionnaire, it is advised to use the Polish utility standard set obtained by means of the—time trade-off method.	Valuing medicines should include gains in length of life and quality of life, as well as adverse effects such as toxicity, which should be included as negative impacts on quality-of-life. SMC prefers generic and validated classification systems which are reliable and appropriate population preference values (choice-based method such as the time trade-off or standard gamble). Ideally, these data will be generated through randomized controlled studies. A higher cost/QALY may be accepted if: more than 3 months survival gain with sufficient quality of life to make the extra survival desirable […] or evidence of a substantial improvement in quality of life (with or without survival benefit). Evidence of a substantial improvement in quality of life (with or without survival benefit); Evidence of a substantial improvement in quality of life (with or without survival benefit).

^a^ No guideline was publicly available. Other sources were used

QoL was considered a relevant endpoint in all jurisdictions. Most guidelines are general and do not mention oncology medicines specifically. In addition, the majority refers that evidence requirements applicable to QoL data are to be the same as for other health effects, e.g. preferably measured in randomized clinical trials. The German guidelines provide some details on how to handle bias from open studies. Some guidelines provide pointers on the potential influence of QoL data in recommendations. German guidelines indicate that for new drugs the demonstration of an added benefit in terms of QoL alone is insufficient when there is no added benefit either in morbidity or mortality. The Dutch guideline refers: ‘*Very little research is undertaken that explicitly focuses on quality of life. However, the added value of a medicine may actually be expressed in the form of an improved quality of life. Consequently, it is always worthwhile mentioning relevant data on this aspect*’.

Some jurisdictions (England, Scotland and The Netherlands) also specify that the well-being of caregivers is relevant. The English guideline states that it is important to “*identify principal measures of health outcome(s) that will be relevant for the estimation of clinical effectiveness. That is, they measure health benefits and adverse effects that are important to patients and/or their carers*”.

The French consensus statement addressed the absence of QoL data, stating ‘*The Commission often bemoans the lack of quality*-*of*-*life data: tools are available in oncology but are difficult to use repeatedly in clinical trials and vary inter*-*individually, which reduces their relevance for deciding between treatments or therapeutic strategies. As cancers become chronic, other tools such as examining patient preference or utility could be taken into account by the Transparency Commission in oncology*’.

### Inclusion of QoL data in REAs

**Fig. 2:**
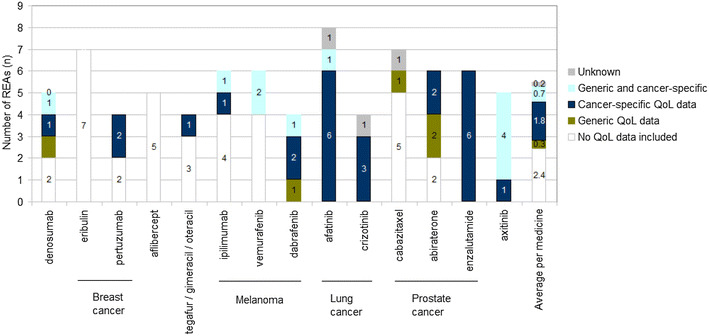
Number of REAs (n) in which quality-of-life data are included per medicine and instrument type

Figure 2 provides an overview of the QoL data included in REAs, per medicine and per instrument type. There are variations across different drugs as to the inclusion of QoL data and as to the instrument being used. For two drugs, no QoL data were included (aflibercept and eribulin) in any of the REAs and for 5 out of the 14 drugs all REAs had QoL data. Also, the type of instrument used (generic vs cancer-specific) varied not only across different medicines but also within the same indication (e.g. cabazitaxel vs. enzalutamide for prostate cancer). On average, cancer-specific QoL data were more frequently included in REAs than generic QoL data.

**Fig. 3:**
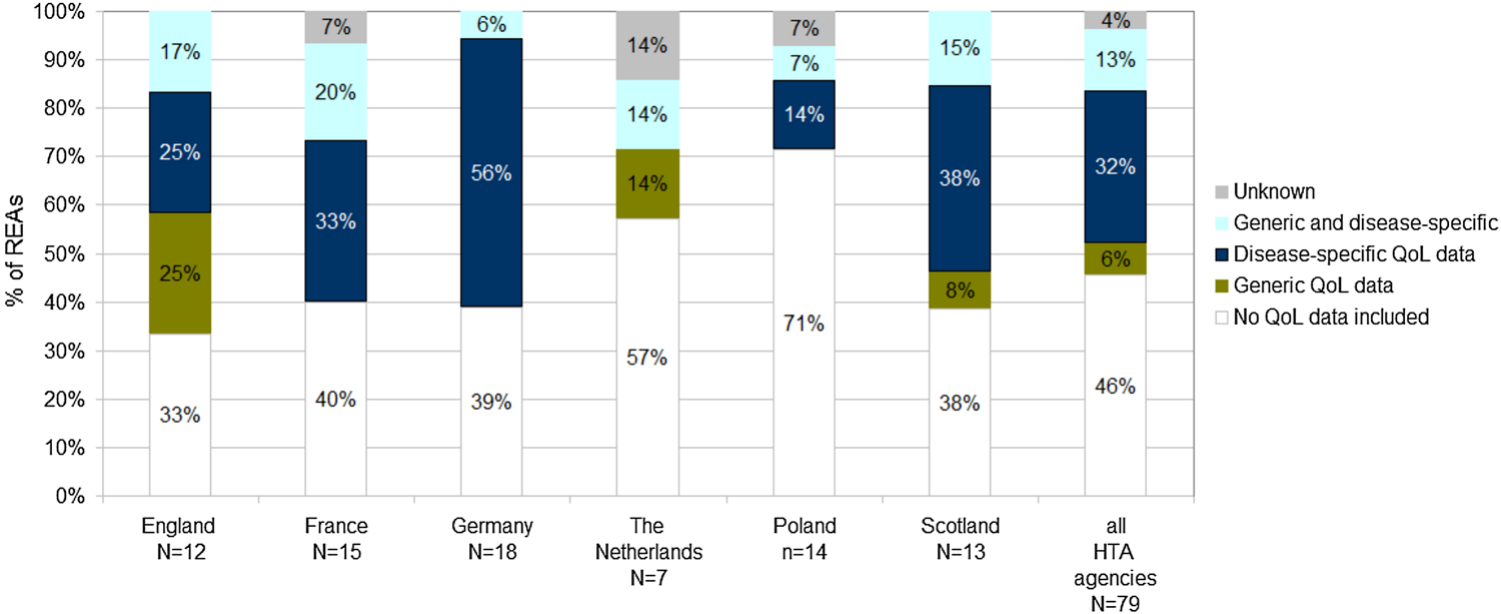
Quality-of-life data and instruments included in REAs per jurisdiction (percentage)

Figure 3 shows the inclusion of QoL data per jurisdiction and type of QoL instrument. The overall percentage of REAs across all jurisdictions in which QoL data were included was 54%; it varied from a lowest of 29% (Poland) to a highest of 67% (England). In what concerns the choice of instrument, Germany stands out with a relatively high percentage of cancer-specific QoL data in its REAs (56%). The Netherlands, on the other hand, only included either generic data or a mix of generic and cancer-specific QoL data.

The most frequently used QoL instruments were the disease-specific FACT questionnaire (included in 24% of the REAs), the EORTC questionnaire (20%) and the generics EQ-5D (10%) and Brief Pain Inventory-Short Form (8%). An overview of QoL instruments retrieved in our sample is presented in Supplementary Table 1.

### Impact of QoL data on recommendation

**Fig. 4:**
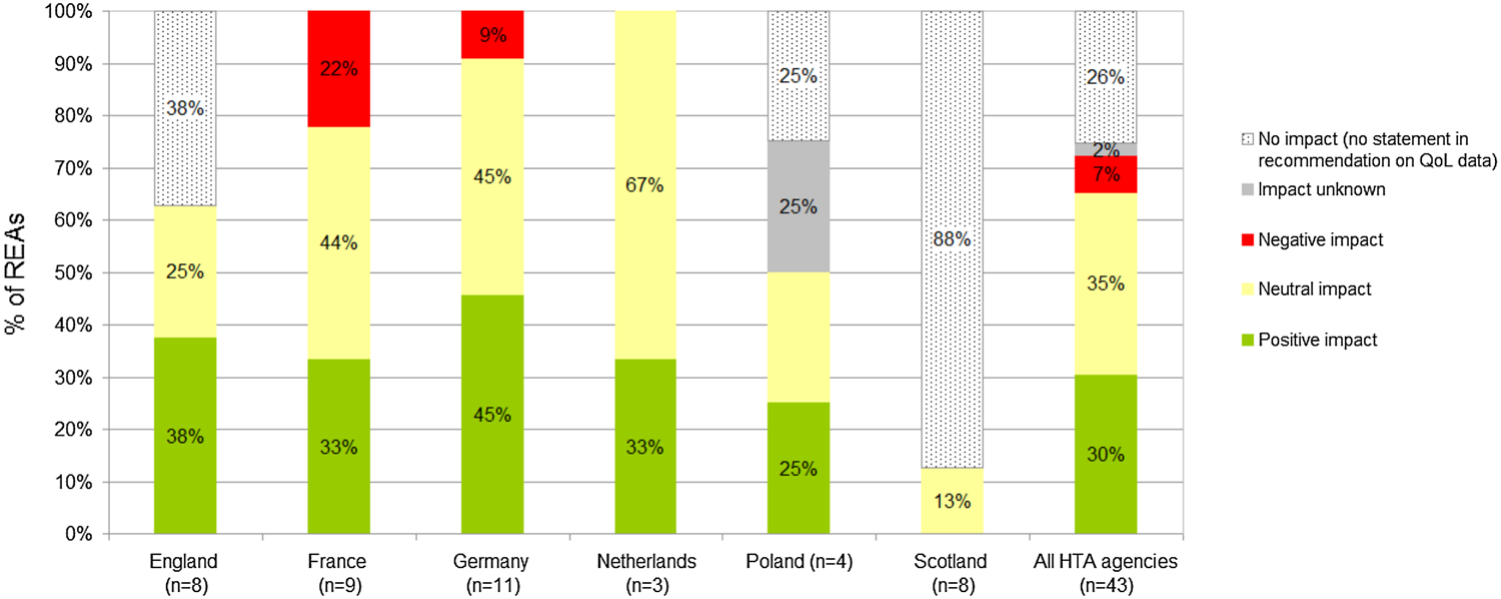
Impact of included quality-of-life data on the recommendation

The impact of included QoL data on the recommendation, per jurisdiction, is provided in Fig. 4. Overall, QoL data did not impact the recommendation in 26% of the REAs (i.e. we did not find a statement on QoL data in the recommendation). Yet this percentage varied substantially at national level from 0% in France, Germany and The Netherlands to 88% in Scotland. The percentage of REAs in which QoL data had a negative impact was relatively low for all jurisdictions (on average 7%). QoL data had a positive or neutral impact on the recommendation in about one-third of the recommendations (respectively, 30 and 35%).

Only the lung cancer drug afatinib received a positive recommendation (‘*hint’* of added benefit in Germany) for a particular subgroup: patients under 65 years of age with a L858R mutation, primarily based on benefits in symptom relief and QoL. In addition, QoL data seem to have had a positive effect on the recommendation for crizotinib (indicated for lung cancer) across multiple jurisdictions, as well as for abiraterone and enzalutamide (both indicated for prostate cancer). Supplementary Table 2 provides some examples of citations categorized as having had a positive or negative impact on the recommendations.

### Association between instrument type and impact on recommendation

A higher percentage of cancer-specific QoL data had a positive impact when compared to generic QoL data or cancer-specific and generic QoL data; however, differences in percentages across instrument types were not significant. The majority of generic QoL data seemed to have had no impact on the recommendation, whereas the majority of cancer-specific and generic QoL data had a neutral impact (Table 2).

**Table 2:** Impact of QoL data on recommendation per type of instrument

	REAs (n)	Positive impact (%)	Neutral impact (%)	Negative impact (%)	Impact unknown (%)	No impact^a^ (%)	Total (%)
Generic QoL data	5	20	20	0	0	60	100
Disease-specific QoL data	25	44	24	12	0	20	100
Generic and disease-specific	10	0	70	0	0	30	100
Unknown	3	33	33	0	33	0	100

^a^No statement in recommendation on QoL data

## Discussion

This study aimed to explore the role played by QoL data in REAs for HTA recommendations of new cancer drugs in European countries.

Whereas guidelines from HTA agencies indicate that QoL data are to be considered a relevant endpoint in the reimbursement decision-making process of new anti-cancer drugs in Europe, evidence from our study suggests otherwise. QoL data were only included in 54% of the REAs reports. In addition, the impact of the included QoL data was limited as no specific statement on included QoL data was identified in one-fourth of the recommendations. Our study also suggests a higher uptake and positive impact of cancer-specific QoL data, when compared to generic QoL data. Moreover, differences exist between countries as to the inclusion and extent of use of QoL data in relative assessments. These differences are indicative of variation across HTA agencies on how they handle and report this type of data.

Other researchers have also reported on the limited availability of (robust) QoL data for oncology medicines [24]. Within our sample of HTA reports, stated reasons for non-inclusion of QoL data were either unavailability (i.e. absence) or lack of robustness. The first cause was applicable to eribulin, with the lack of QoL data in the pivotal EMBRACE study being highlighted in the English, French, Dutch and Scottish assessments. QoL data were considered to be insufficiently robust in the German assessments of abiraterone and pertuzumab. Even though the weakness of the QoL evidence is mentioned in several recommendations (e.g. eribulin, abiraterone, aflibercept and pertuzumab), we learnt during the expert panel consultation that this shortcoming does not generally negatively impact the final HTA recommendation. Results from a contemporary study indicate that data on other endpoints, such as overall survival and progression-free survival, play a more decisive role in the recommendation than QoL data [23]. De facto, within our dataset, only one drug—afatinib—received a positive recommendation for a specific subgroup due to its beneficial effects on QoL and symptom relief.

There is evidence supporting the inclusion of patient-reported outcomes, including health-reported QoL, in regulatory product approvals [15, 17–19]. Vodicka et al. investigated the entries in the US clinicaltrials.gov register between 2007 and 2013 and reported an increase in the collection of patient-reported measures from 2009 onwards, particularly oncology drug trials [25]. While this trend might, at first glance, suggest future improvements, there are no guarantees that the endpoint data will be duly collected, reported and of sufficient quality to meet drug regulators’ requirements and HTA agencies’ needs. Such difficulties in retrieving and valuing patient-reported outcomes within HTA assessments have been reported by Triggs and Howells, who looked into NICE recommendations for new pharmaceutical products over 2014 [26]. They concluded that guidance on the use of patient reported outcomes for clinical-effectiveness assessments was vague and thus compliance was very low. They added that a stringent approach was needed when assessing patient-reported outcomes data, to ensure accurate measurement of treatment effectiveness.

In those jurisdictions where HTA guidelines indicated a QoL instrument preference (England, Poland and Scotland), generic QoL data seemed to be favoured. Nevertheless, our study indicates that cancer-specific QoL data seem to have greater impact on recommendations than generic QoL data. This confirms previous results from other research on REA methods across 29 jurisdictions, which showed that disease-specific QoL measurements were more widely accepted [21]. The guideline produced by the European Network for Health Technology Assessment (EUnetHTA) of the use of QoL data in REAs refers that the choice of the QoL measure is dependent on the purpose of the REA and the decision-making context, but that consensus on QoL evidence is often lacking due to variations in context [11]. They recommended that REAs aimed at coverage decisions should include both a disease- or population-specific measurement as well as a generic QoL measure, so that the impact of a disease on daily life can be adequately captured. Within our dataset, this mix of disease and generic measurements was only available in 17% of the REAs reports.

Yet, Cleemput et al. also emphasize that recommendations informing decisions on resource allocation across various indications should primarily be based on generic QoL data, as only generic instruments enable comparisons between multiple indications and intervention types [11]. They indicated that within a given indication, disease-specific QoL data may be suitable, but recommend, in addition to the disease-specific measure, the use of complementary generic QoL data to ensure that all potentially relevant dimensions are included.

Measuring QoL is also relevant to grasp a new drug’s safety profile. According to Trask, the inclusion of health-related quality of life in clinical trials can help identify which treatment-related symptoms are having a negative impact on patients, sometimes even before the QoL changes observed are noted as adverse events [7]. Recent pharmacovigilance legislation in the European Union also encourages the inclusion of patient-reported data in the assessment of a drug’s benefit-risk balance [27, 28].

While the HTA agencies identified in our study considered QoL to be a relevant endpoint to be taken into account during relative effectiveness assessments, they also reported concerns about the methodological constraints of QoL data collection and their subsequent quality. Further steps needed to improve data collection would include reducing providers’ inexperience with QoL instruments, tackling methodologic barriers such as the limitations of QoL instruments in detecting clinically meaningful changes and addressing feasibility and logistic difficulties such as time constraints [7]. HTA bodies are in a key position to proactively stimulate better collection of QoL data by establishing standardized evidence requirements.

The general limitations of this study include the restricted number of European HTA jurisdictions; the variability in drugs assessed per jurisdiction; as well as challenges faced in the interpretation of value statements from HTA reports and the fact that our study’s methods and results somewhat simplify real-world decision making. We have opted to focus on the role of QoL data in REAs, and not on pharmacoeconomic assessments, as relative effectiveness is the most commonly shared criterion for pricing and/or reimbursement recommendations in EU jurisdictions [21]. Nevertheless, it is very likely that QoL would have a more prominent role in pharmacoeconomic assessments of oncology drugs given its relevance in utility analysis of quality-adjusted life years. Finally, this research was restricted to oncology medicines and it remains unclear whether our findings would be applicable to other indications. Recent research has shown that the type and frequency of patient-reported outcomes used in clinical trials are largely dependent of the disease being studied [29].

## Conclusion

There seems to be a lack of (robust) QoL data in REAs for oncology drugs. Yet apparently, this current absence of robust QoL data does not impact the recommendations. Further collaboration is needed to promote the use of robust QoL data and to map strategies to improve the use of this patient-centred outcome in future reimbursement decisions. HTA bodies are in a key position to proactively stimulate better collection of QoL data by establishing standardized evidence requirements for valid and reliable QoL data to be used in REAs. This could potentially encourage pharmaceutical companies to incorporate robust QoL measures in their clinical research and subsequently provide regulatory agencies and HTA institutions with more complete dossiers for assessment.

## Electronic supplementary material

Below is the link to the electronic supplementary material.

Supplementary material 1 (DOCX 65 kb)

Supplementary material 2 (DOCX 16 kb)

## Footnotes

1 There are 28 EU member states; however, UK was divided into two HTA jurisdiction (England and Scotland) due to extensive experience of the national institute for health and clinical excellence (NICE) and the Scottish Medicines Consortium (SMC).
