# Supplementary material for: The impact of quality-of-life data in relative effectiveness assessments of new anti-cancer drugs in European countries
Source: Qual Life Res. 2017 Apr 11;26(9):2479–88. doi: 10.1007/s11136-017-1574-9 (PMC5548837; doi:10.1007/s11136-017-1574-9)
Supplement: Supplementary file 1 — Supplementary material 1 (DOCX 65 kb) [file 11136_2017_1574_MOESM1_ESM.docx]

**Supplementary Table 1. Descriptive table of the quality of life instruments retrieved in our sample**

| Name of QoL instrument | # of REAs in which was included (total n=79) | Medicines |
| --- | --- | --- |
| FACT-(B, FKSI-15, FKSI-DRS, G, Ga, M, P) | 18 | tegafur/gimeracil/oteracil (FR), denosumab (NL,PO), abiraterone (FR), vemurafenib (GE,SC), axitinib (GE,UK,SC, FR) pertuzumab (FR, SC), enzalutamide (GE, GE, UK, FR, SC, PO) |
| EORTC-QLQ-(BR23, C30) | 15 | ipilimumab (GE, FR), axitinib (PO), crizotinib (GE, UK), dabrafenib (GE,FR,SC), afatinib (GE, GE, GE, GE, UK, FR, SC) |
| EQ-5D | 8 | Denosumab (UK,NL) axitinib (UK,SC,PO), afatinib (UK), , dabrafenib (UK,FR) |
| EQ-VAS | 2 | vemurafenib (GE), afatinib (UK) |
| BPI-SF | 6 | Denosumab (NL), abiraterone (NL,EN, SC), enzalutamide (GE,GE) |
| Pain response* ^†^ | 2 | Cabazitaxel (UK, NL) |
| SF-36 | 1 | Ipilimumab (FR) |
| Pain progression ^†^ | 1 | Cabazitaxel (UK) |
| Time to detoriation ^†^ | 1 | Crizotinib (SC) |
| Time to developing pain  ^†^ | 1 | Denosumab (UK) |
| Analgesic use ^†^ | 2 | Denosumab (UK,NL) |

* Measured with present pain intensity score on the McGill-Melzack scale

† These are also considered to be morbidity-related patient reported outcomes.
