# Supplementary material for: The impact of quality-of-life data in relative effectiveness assessments of new anti-cancer drugs in European countries
Source: Qual Life Res. 2017 Apr 11;26(9):2479–88. doi: 10.1007/s11136-017-1574-9 (PMC5548837; doi:10.1007/s11136-017-1574-9)
Supplement: Supplementary file 2 — Supplementary material 2 (DOCX 16 kb) [file 11136_2017_1574_MOESM2_ESM.docx]

| **Supplementary Table 2 – Examples of the positive and negative impact of quality**  **of life data excerpts included in HTA recommendations** | | | |
| --- | --- | --- | --- |
| **Positive Impact** | | | |
| **Medicine** | **Indication** | **HTA body and jurisdiction** | **Citation** |
| abiraterone | Prostate cancer | NICE  England | *“The patients’ quality of life deteriorates less under treatment than with placebo.” “The Committee also noted that patients receiving abiraterone were more likely to experience an improvement in symptoms, including pain, functional status and fatigue.”* |
| crizotinib | Prostate cancer | HAS  France | *“In view of the available clinical data, in particular a study versus chemotherapy with docetaxel or pemetrexed showing an absolute increase of 4.7 months in progression-free survival in favour of XALKORI, with a significant improvement in objective response rate as well as quality of life, a moderate additional impact in terms of morbidity, mortality and quality of life is expected in patients treated with XALKORI as a second-line therapy compared with chemotherapy.”* |
| enzalutamide | Non-small-cell lung cancer | IQWIG  Germany | *“FACT-P: Outcome category: non-serious/non-severe symptoms/late complications CI< 0.80 Added benefit, extent: considerable”*  *BPI-SF: Added benefit, extent: non-quantifiable.”* |
| **Negative Impact** | | | |
| afatanib |  | IQWIG  Germany | *“Negative effects: Health-related quality of life (in each case “hint”):  role functioning (≥ 65 years)  improvement; “minor”* |
| enzalutamide | Non-small-cell lung cancer | HAS  France | *“The fragmented quality of life data cannot quantify the impact of XTANDI on the quality of life of the patients treated.”* |
| tegafur / gimeracil / oteracil | Gastric cancer | HAS  France | *“Available data do not show the impact of TEYSUNO in terms of a reduction in morbidity and mortality or the improvement in quality of life; therefore it is not expected that this medicinal product will have an additional impact compared with current treatments.”* |
